# Supplementary material for: Intrauterine Zika virus infection of pregnant immunocompetent mice models transplacental transmission and adverse perinatal outcomes
Source: Nat Commun. 2017 Feb 21;8:14575. doi: 10.1038/ncomms14575 (PMC5321801; doi:10.1038/ncomms14575)
Supplement: Supplementary Information — Supplementary Figure [file ncomms14575-s1.pdf]

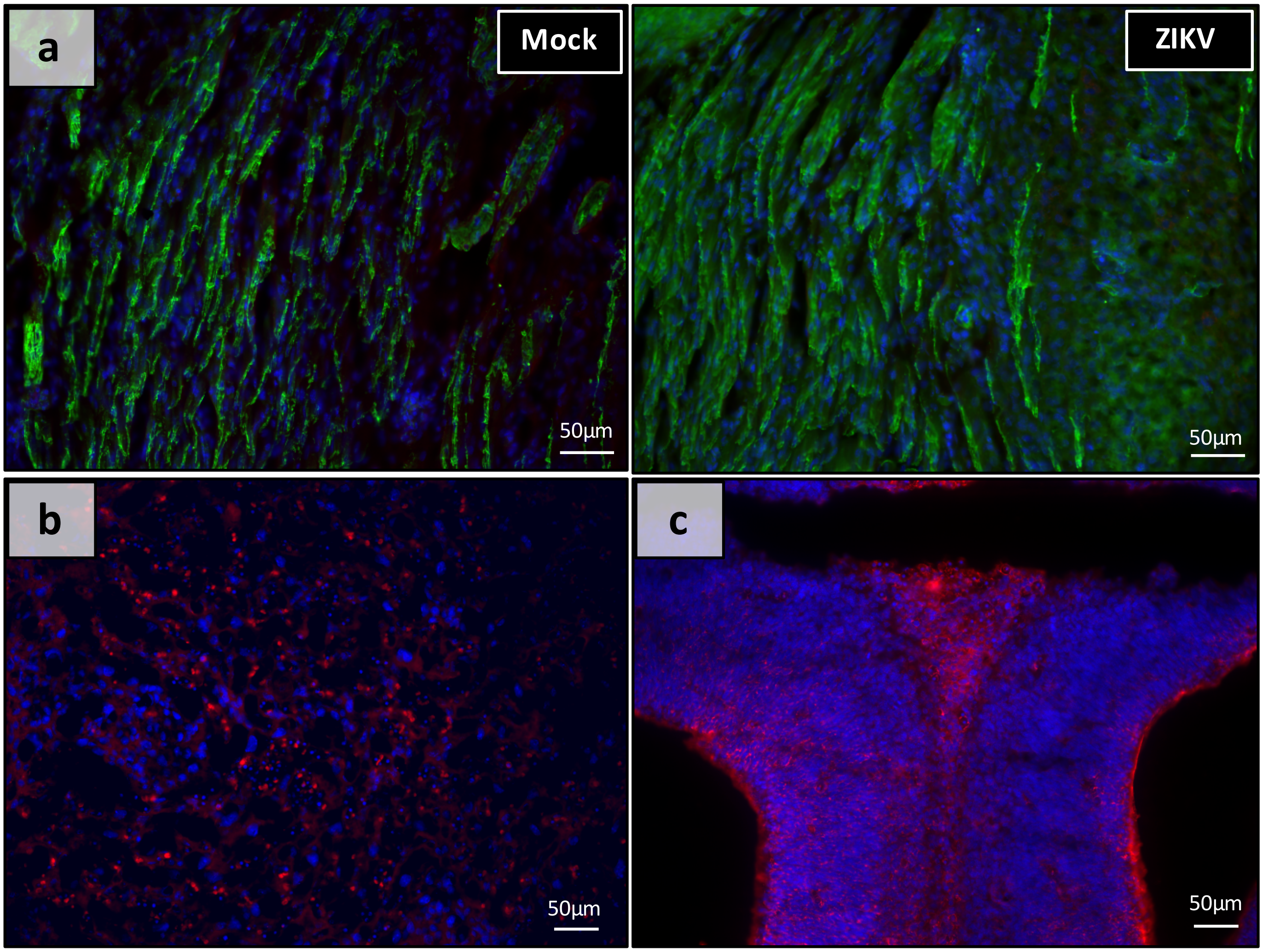

Supplementary Figure 1. ZIKV antigen in the uterine myometrium, placenta, and fetal brain. Pregnant CD1 dams underwent a mini-laparotomy in the lower abdomen for intrauterine (IU) inoculation of ZIKV (1968 Nigeria) or vehicle at embryonic day 10. Uterine horns were harvested 48 hours post-inoculation (hpi) and placentas and fetal brains were harvested 96 hpi for immunohistochemical analysis. (**a**) Fluorescent immuno-staining of ZIKV (red) and α-actin (green) with 4'6-diamidino-2-phenylindole (DAPI, blue) to label nuclei in mock-infected (left panel) and infected (right panel) uterine horns. (**b**) Fluorescent immuno-staining of ZIKV (red) with 4'6-diamidino-2-phenylindole (DAPI, blue) to label nuclei in infected placentas collected 96 hpi. (**c**) Fluorescent immuno-staining of ZIKV (red) with 4'6-diamidino-2-phenylindole (DAPI, blue) to label nuclei in infected fetal brains collected 96 hpi. Scale bar, 50 µm.
